# Participant Evaluation of a Multi-disciplinary Oncology Preceptorship Training Program for Oncology Health Professionals from Kumasi, Ghana

**DOI:** 10.1007/s13187-024-02417-w

**Published:** 2024-03-20

**Authors:** Kenneth W. Merrell, Thomas Okpoti Konney, Osei Acheamfour, Joseph Lucido, Abena Yeboah Aduse-Poku, Amanika Kumar, Mavis Bobie Ansah, Adu Tutu Amankwa, Dean Shumway, Fred Kwame Awittor, Augustina Badu-Peprah, Lionel Aurelien A. Kankeu Fonkoua, Andrea E. Wahner Hendrickson, Ernest Boakye, Ernest Kwasi Adjei, Ishmael Kyei, Katie Kemper, Miranda Rank, Prema P. Peethambaram, Kathryn Spangenberg, Kasie Sorenson, Miranda Hearrold, Allison Garda, Rondell Graham, Karen Lang, Joseph Adom, Rita Achiaa, James Jakub, Bismark Dwobeng Amo, Ernest Osei-Bonsu, Rolando Camacho, Eric Clement Desmond Kotei Addison

**Affiliations:** 1 Department of Radiation Oncology, Global Bridges, Mayo Clinic, Rochester, MN USA; 2 Komfo Anokye Teaching Hospital, Kumasi, Ghana; 3 Department of Radiation Oncology, Mayo Clinic, Rochester, MN USA; 4 Department of Obstetrics and Gynecology, Mayo Clinic, Rochester, MN USA; 5 City Cancer Challenge, Kumasi Cancer Registry, Kumasi, Ghana; 6 Quitt Health Care Limited, Kumasi, Ghana; 7 Department of Medical Oncology, Mayo Clinic, Rochester, MN USA; 8 Ernphil Laboratory and Diagnostic Services, Kumasi, Ghana; 9 Global Bridges, Mayo Clinic, Rochester, MN USA; 10 American Cancer Society, Washington, DC USA; 11 Department of Laboratory Medicine and Pathology, Mayo Clinic, Rochester, MN USA; 12 Peace and Love Hospital, Kumasi, Ghana; 13 General Surgery, Mayo Clinic, Jacksonville, FL USA; 14 City Cancer Challenge, Geneva, Switzerland; 15 Kwame Nkrumah University of Science & Technology, Komfo Anokye Teaching Hospital, Kumasi, Ghana

**Keywords:** Multidisciplinary team, Clinical skill development, Cancer training, Global health disparities

## Abstract

A critical shortage of skilled healthcare workers is a primary cause of disparate global cancer outcomes. We report participant evaluation of a multidisciplinary preceptorship program. In collaboration with the city of Kumasi, Ghana, Mayo Clinic and the City Cancer Challenge hosted a preceptorship program for comprehensive multidisciplinary breast and cervix cancer training. A total of 14 healthcare workers from Kumasi received two weeks of training at Mayo Clinic in November and December 2021. Each participant and preceptor were requested to complete an anonymous post-participation survey. Of the 14 trainee participants, 10 (71%) completed the survey. All respondents found the program “valuable and applicable to their clinical practice.” Ninety percent reported they were able to “review effective and critical elements in the development and expansion of the multidisciplinary team” and able to “solve practical clinical cases as a team”. General themes of satisfaction included: (1) organization and administration, (2) clinical observations and demonstrations, (3) guidelines development, and (4) recognizing the central importance of cultivating a team-based approach. Of the 40 preceptors, 16 (40%) completed the survey. All respondents reported they felt the training would meaningfully “influence patient care in Ghana”, that participation “added value or joy to their clinical practice,” and all wished to “participate in future preceptorship programs”. After a focused two-week program, trainees reported high satisfaction, usefulness from observing specialized cancer care, and value in closely observing a multidisciplinary oncology team. Preceptors reported the experience added joy and perspective to their clinical practice and wished to participate in future programs.

**Supplementary Information:**

The online version contains supplementary material available at 10.1007/s13187-024-02417-w.

## Background

Cancer is one of the leading causes of premature death worldwide with three-quarters of the total burden occurring in low- and middle-income countries (LMIC) [1–8]. Current estimates for Africa indicate approximately 1.0 million new cancer cases and 700,000 cancer deaths yearly [1]. As more than three quarters of cancer cases in Africa are advanced or metastatic, the most common cancers of the breast, cervix, and prostate are more than twice as deadly than in North America due to gross disparities in healthcare resources [4, 5, 8, 3, 9]. These disparate outcomes are primarily a result of both severe limitations in physical and material resources and the limited number of highly trained healthcare workers in all disciplines and levels of oncology.

For much of Africa, trainee enrollment has outpaced retention and training of medical faculty to serve as mentors and advisors. In Tanzania, for example, the annual approved medical student positions increased from 55 in the 1990s to over 1500 in the 2015–2016 academic year [10]. However, of the five medical schools with available faculty information, there are only 366 appointed faculty members, of which nearly two-thirds are junior. Oncology is one of the least populated disciplines with fewer than 10 faculties. Other reports also highlight the challenging situation of trainees greatly outnumbering the senior faculty and the need for more comprehensive training programs [11]. A 2019 survey of general medical providers in Rwanda revealed that less than one third of respondents had ever attended a continued medical or professional development program[12]. This is despite nearly all (99%) desiring participation in such a program to continue their medical education. The survey also showed a desire for training programs with case-based learning and group discussions among physicians with the specific desire to learn algorithms to help guide cancer diagnosis [12].

In 2018, the city of Kumasi, Ghana, in partnership with the City Cancer Challenge (C/Can), conducted a comprehensive assessment of cancer care capacity and needs involving nearly 200 health professionals in all specialties and 38 health institutions from both the public and private sectors. Among the main challenges identified by the technical groups in the city were 1) the need to expand the multidisciplinary approach to cancer care and 2) a lack of clinical management guidelines adapted to the available resources. As a response to these challenges, the technical groups in Kumasi, supported by C/Can and advisors, designed a project to develop guidelines on the management of patients diagnosed with the most common and curable cancers in the city, starting with cervix and breast. Several capacity building projects were identified to support the implementation of these projects, including a scientific visit, or preceptorship program, to a foreign academic center to be executed in 2021.

Here we report the results of a participant and preceptor satisfaction survey of a Mayo Clinic preceptorship program aimed at developing a multidisciplinary care team co-developed by Mayo Clinic, C/Can, and health professionals from city of Kumasi and Komfo Anokye Teaching Hospital (KATH), located in the Ashanti Region Ghana.

## Methods and Materials

In 2018, Mayo Clinic initiated a collaborative medical training and preceptorship program in medical physics and clinical radiation oncology with healthcare workers from Kumasi, Ghana. The primary objectives were to develop capacity for advanced radiation therapy planning and delivery, and to improve clinical and medical oncology knowledge through a preceptorship experience at Mayo Clinic. Five healthcare workers from KATH came to Mayo Clinic for a period of 2 to 8 weeks to receive the training. The on-site training was followed with a continued medical education program via an online, virtual platform. In addition to clinical capacity building, the preceptorship helped refine the multi-disciplinary team structure including team dynamics, culture, and leadership. Prior to the visit in 2019, the KATH Oncology Directorate had just begun implementing the basic 3-D conformal radiation techniques with their newly installed linear accelerator at the facility. Two years later, the KATH team now treats hundreds of patients a year with high quality and standardized advanced radiotherapy.

Kumasi was the first African city to join the C/Can initiative in 2018. Now operational in 11 cities across Africa, Asia, Eastern Europe, and Latin America, C/Can leverages the unique value of cities as enablers in a health systems’ response to cancer that prioritizes the needs of end users (patients, their caregivers and families, and healthcare providers). Cities are supported through an engagement process whereby local stakeholders lead a staged city-wide process over a 2- to 3-year period to assess, plan, and execute locally adapted cancer care solutions [13].

Based on the past success in design and implementation of a preceptorship program, Mayo Clinic Cancer Center’s outreach program Global Bridges Oncology (GBO), was selected in 2021 to support the city of Kumasi, Ghana, and C/Can with a multidisciplinary preceptorship program at Mayo Clinic. A total of 14 oncology specialists from Kumasi representing all facets of multidisciplinary cancer care were selected to travel to Mayo Clinic for the program. Participants were separated into two groups based upon clinical focus: breast cancer group and cervix cancer group. A total of 6 participants in the breast cancer group participated in the program from November 1–12, 2021, and 8 participants in the cervix cancer group participated from November 29–December 10, 2021.

Each participant was assigned an individual preceptor from the same field or discipline to support the participant’s visit and aid in designing a unique and informative program in accord with the program’s objectives, including identifying other faculty to participate in the training. In all, more than 80 cancer center members, including leadership, administrators, and 40 faculty members provided training, either directly with clinical instruction, or indirectly through administrative support. Each participant from Kumasi joined activities of the department or division of their respective specialty, and applicable other specialties, for the duration of the visit. For example, the breast surgeons spent time embedded within the cancer clinic, operating room, pathology lab, and radiology suite to review breast cancer screening and treatment initiatives. Outside of the clinic and shadowing experience, participants were invited to join in multi-disciplinary tumor boards, chart rounds, grand rounds, and other didactic sessions. Participants were also invited to provide lectures and other educational activities to Mayo Clinic staff for the education of the host institution.

At completion of the program, participants and preceptors received separate online surveys to anonymously provide a review of the program, including both strengths and areas for improvement. The survey was administered through the Qualtrics XM (Qualtrics, Provo, UT) operating system and consisted of a 5-point Likert scale and yes/no questions (Supplemental Table 1). Level of agreement was indicated by response options consisting of “strongly agree”, “somewhat agree”, “neither”, “somewhat disagree” and “strongly disagree”. An additional 3 items were open-text entries in which respondents could write their own answers.

## Results

### Preceptorship Program Participants

A total of 10 out of the 14 program participants completed the survey. A total of 4 participants were female (28%) and 10 participants had a medical or equivalent degree, 2 had nursing degrees and 3 had master’s level physics degree. Disciplines represented in the preceptorship program include Radiation Oncology (*n* = 2), Medical Oncology (*n* = 1), Gynecologic Surgery (*n* = 1), Breast Surgery (*n* = 1), Radiology (*n* = 2), Pathology (*n* = 2), Medical Physics (*n* = 3), and Oncology Nursing (*n* = 2).

**Fig. 1:**
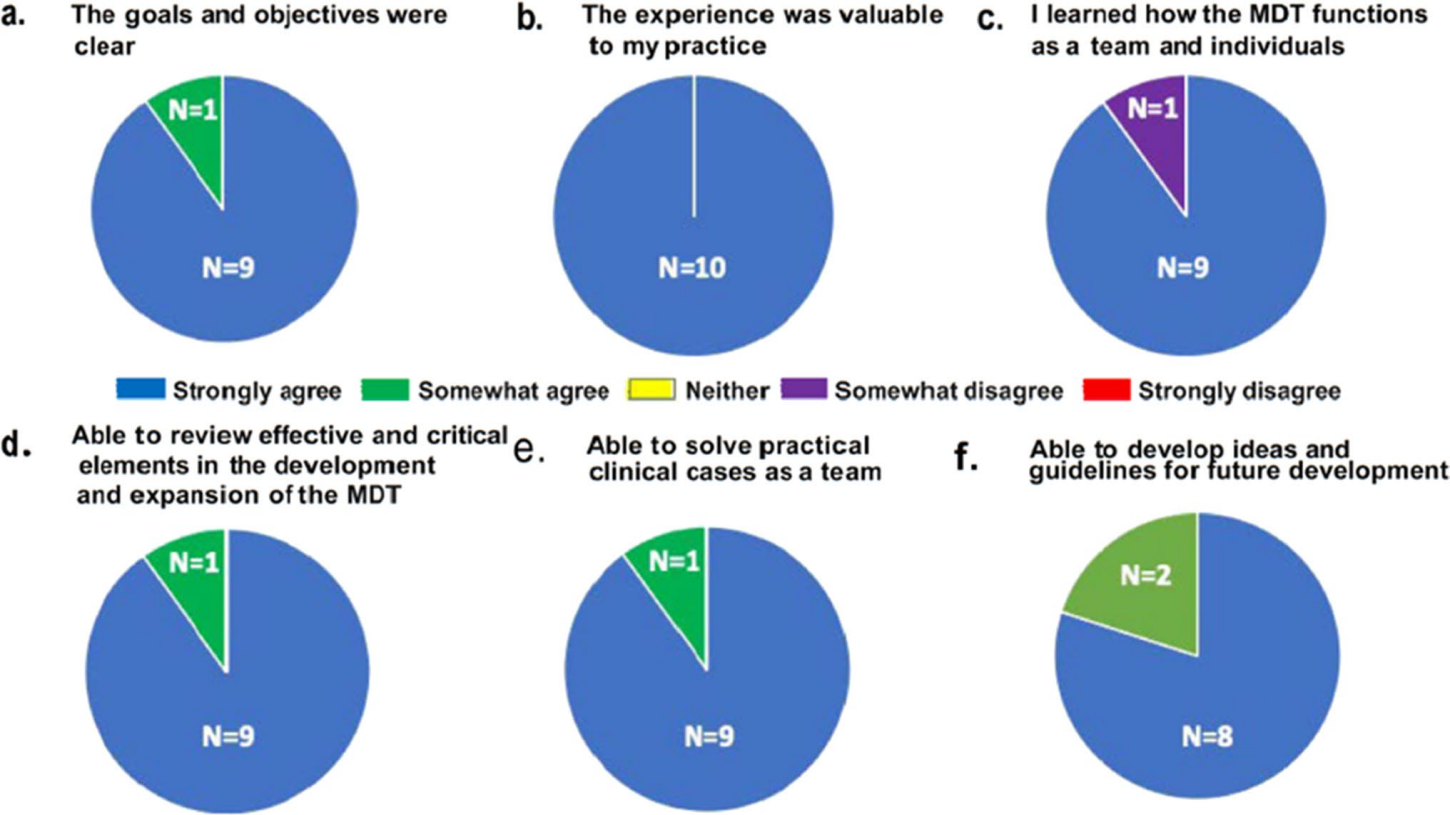
Survey assessment of participant experience. Pie chart using 5-point Likert scale to report trainee participant experience and satisfaction related to the program goals

All 10 respondents indicated the visit to Mayo Clinic was valuable and applicable to their clinical practice. Out of the 10 respondents, 1 participant “somewhat disagreed” with the statement, “I was able to learn how the multidisciplinary team treating breast or cervix cancer at Mayo Clinic function as a team and as individual specialists” and indicated they were not able to participate in a multidisciplinary tumor board. However, 90% of respondents indicated they were able to “review effective and critical elements in the development and expansion of the multidisciplinary team” and able to “solve practical clinical cases as a team”. The percent reporting, I “strongly agree” or “somewhat agree” with having achieved success in the ability to “develop ideas and guidelines” was 90% and 10%, respectively (Fig. 1).

Respondents were asked to share their qualitative feedback on the program (Table 2). General themes reported satisfaction with topics related to: (1) organization and administrative support, (2) clinical observations and demonstrations, (3) guidelines development, and (4) networking with preceptors. When asked about the most important takeaways from the trip, comments focused on how an effective multidisciplinary team may improve patient care and outcomes and highlighted the unique components of this team. When asked for areas of improvement, the most common request was to increase the stay from two weeks to longer. Other areas for improvement included identification of trainers with specific interest in global oncology (Table 1).

**Table 1:** Participant Written Feedback

Theme	Comments
Organization and administrative support	• The trip was well organized, and faculty were aware of our mission • 1. Warm reception 2. well planned itinerary 3. Ever present support to navigate clinics 4. Constant checking on our well beings. Everything else so well organized and appreciated
Clinical observations and demonstrations	• Patients are the number priority and hence are treated with respect and necessary courtesies. Knowledge team of consultants who are always ready to teach. Great teamwork • I learned new things at the chemo unit that is advising the lymphoedema patient on salt intake. Educating the newly diagnosed patient, radiation skin management and many others • VMAT treatment planning platform, HDR treatment, introducing TBI, Medical Physics QA, LDR Prostate seed, on-line linear accelerator simulation for physics, biomedical engineers, and doctors • How teamwork helps in better management of patients. Radiologists in Ghana should be encouraged to subspecialize which helps in writing better reports and subsequently good management of patients. Ability to work with different radiologist to see how they work. • Consultation with other disciplines and among colleagues was super.
Guideline Development	• Cancer care is multidisciplinary approach. There should be the need for adequate health financing. Research plays critical role in continuous quality improvement in cancer care. • I am confident in giving education on management on lymphoedema in cancer management, radiation skin management and client education so i will be organizing workshops for the nurses at my facility • Transformation of knowledge acquired • There is a lot that can be done for patients with cancer irrespective of stage of the disease or age of the patient • MDT is highly effective and i believe the lessons learnt here will help us improve on our MDT and service delivery • Strong teamwork by a multidisciplinary team enhances Patients’ outcome
Networking with preceptors	• The trip also made it possible for me to establish link with some management members which will likely help in future collaborative work
Areas for Improvement	• So far so good • The multiple disciplinary team meeting • More time • Length of interactions • To get fewer and committed people to shadow. • There is the need to identify interested focal persons in the various specialties who should manage the shadowing processes. • For radiology four weeks would have been ideal instead of 2 weeks

### Mayo Clinic Preceptors

**Fig. 2:**
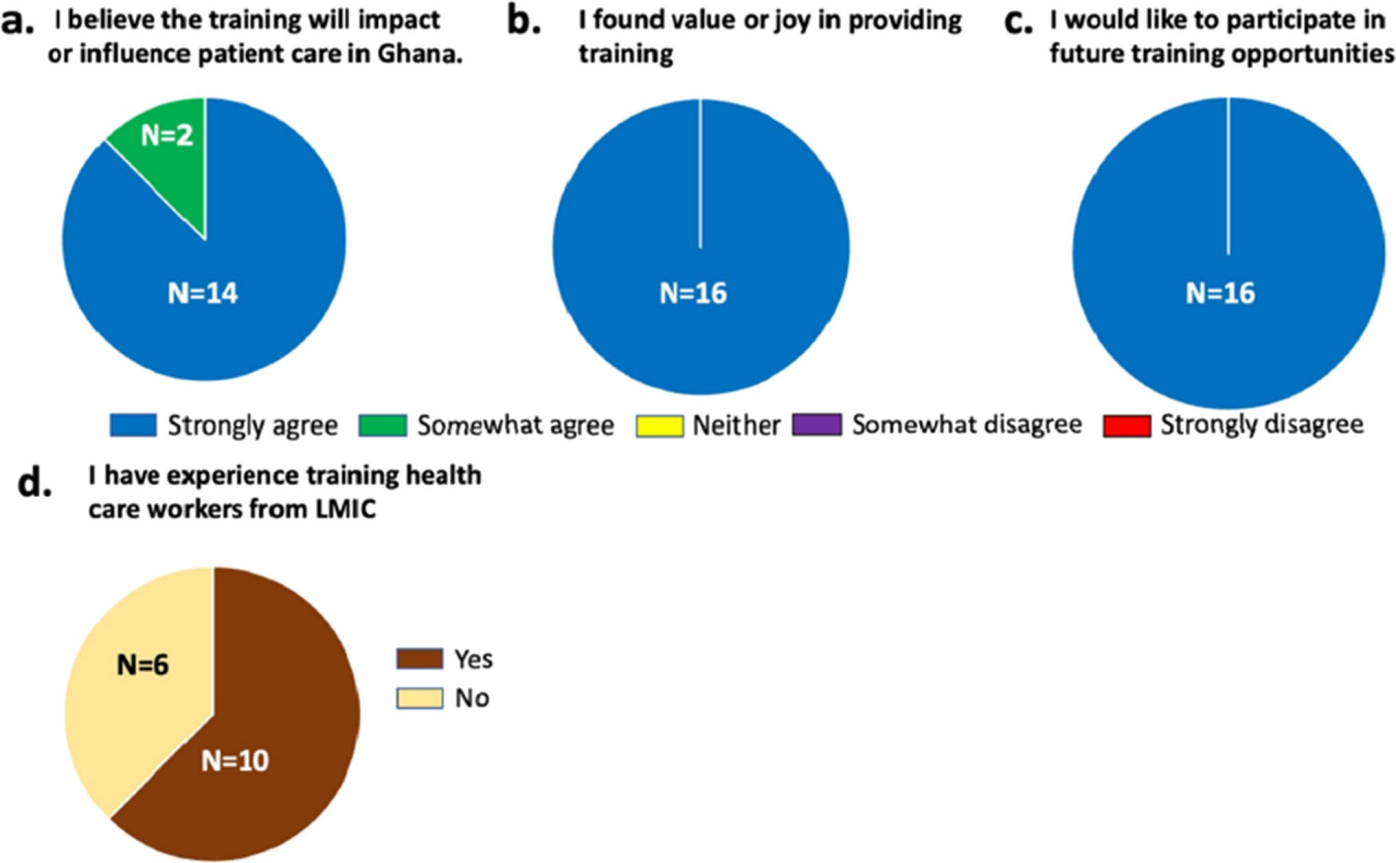
Survey assessment of preceptor experience. Pie chart using 5-point Likert scale yes/no questions to report preceptor participant experience and satisfaction related to the program goals

Out of the 40 Mayo Clinic preceptors who participated in the program, a total of 16 completed the post-visit preceptorship survey (Fig. 2). A total of 62.5% (*n* = 10) of respondents had prior experience providing training to healthcare workers from LMIC. All respondents (*n* = 16) agreed with the statement “I believe that the training I provided will impact or influence patient care in Ghana” with 87.5% (*n* = 14) indicating they “strongly agreed”. All respondents indicated they would like to participate in future preceptorship training programs, and all indicated the experience added value or joy to their clinical practice.

Preceptor comments related to positive, negative, and areas for program improvements are shown in Table 2. General themes related to the positive aspects of the program included enjoyment from: (1) collegial interactions, (2) strong participant enthusiasm, (3) the opportunity to learn of new cultures and healthcare systems, and (4) reflection on one’s own practice with greater appreciation. General themes related to negative aspects or areas for improvement included (1) none, (2) request for more opportunities to participate in longitudinal follow-up of outcomes, (3) challenges in balancing busy schedule and teaching, and (4) the need to lengthen the duration of the program.

**Table 2:** Preceptor Written Feedback

Theme	Comments
Positive Aspects	• I am amazed by the dedication and gratitude that comes from the Ghana group. They are outstanding individuals and truly want to find ways to help their patients. • Learned to look at my practice from a new perspective. • They were so excited to learn, it made it fun! • The Ghana team is very receptive to our insight and knowledge, making training productive as well as enjoyable. • I received just as much joy from helping the professionals from Ghana as they did be here. They were wonderful to work with and they were very appreciative of what we did for them. It also made our group better because of them. What an amazing experience for everyone involved. I would do it again and again, no question! • I couldn’t work with professionals directly, but members of my team did, and they really enjoyed working with them. • Great collegial interaction with a Radiologist who was very keen to learn and collaborate. Very impressive leadership and pioneer work! • A total joy • The participants were very knowledgeable and asked excellent questions. • The nurse that I had the pleasure of spending time with was extremely pleasant and open to any education / recommendations. She took lots of notes and made many comments about how she could use what we do here in Ghana. She also provided a lot of insight into their culture and medical care. • I was able to work with a nurse from Ghana and the experience was incredible. She explained the standard of care in her home country, and it was extremely eye opening. It was exciting to share our practice with her! • Extremely excited to learn and grow the practice, improve care delivery to patients. Fantastic people and colleagues, excited to work with them in the future • I was forced to thing about clinical issues from a new perspective, which forced me to think outside the box. I now approach aspects of my practice with a greater appreciation of the big picture.
Negative Aspects	• The hardest part is getting dedicated time to teaching. It felt like we were multitasking for most of the time while they were with us. It would have been nice to have time to focus solely on teaching. • The resources we have for educators has been slim but is getting better. I wish we had more on staff to help accommodate this type of situation. We did the best we could with what we had, but I do feel like it could have been better with more help for teaching in our group. • Time to train. I strongly believe in the effort that is being made to improve access and quality of treatment for patients in Ghana. I think the department is supportive of this effort also given the strategic plan but like the last visit, my team did not have adequate staffing to provide the best experience possible for the professionals. • It was difficult to organize and coordinate with the visitors • Unfortunately, most of my clinic days were spent seeing non-gyn/breast related patients. Most of the patient scenarios were not directly relevant to their practice e.g. neoadjuvant locally advanced pancreas, proton therapy
Areas for Program Enhancement	• Looking forward to more opportunities to improve cancer treatment in Ghana • Thank you for letting me be a part of this amazing experience. I have gained some new friends and a new perspective on life and what the rest of the world is dealing with and the challenges they must endure. It makes you appreciate what you have and have high hopes they will have the same thing. • I think we need to prioritize time and effort for staff to be able to teach the professionals if/when they return. We need more resources to accommodate and improve upon the highly valuable experience. • I wish I could have carved out more time to spend with the group, and specifically the surgical members. • I would have very much enjoyed doing that and feel back I was unable to. • I would love to see this program expand! • Ability to do longitudinal teaching/care and collaboration. Two weeks is just too short! • I would like to have been able to know the schedule a little more in advance so I could coordinate with my team members to facilitate getting the right people into the right meetings.

## Discussion

Integration of academic programs from high-income countries (HIC) into LMIC are critical to support advances and improve disparate cancer outcomes. Past reports highlight the critical need for training programs for healthcare workers from LMIC, specifically SSA. An audience survey of African cancer specialists at the Breast Cancer in Africa Symposia showed the two highest priorities and needs as (1) the development of educational and training programs and (2) the sharing of medical supplies [14]. However, despite a strong desire and need for sustainable international training programs, very few are available, and only a small minority of healthcare workers may have the opportunity to advance their medical education. A 2019 survey showed a strong desire for continued education but a severe lack of opportunities [12]. Challenges with HIC integration into SSA programs are several but generally include sporadic involvement without sustainability, programs not tailored to needs of the healthcare workers or region, and timelines that do not match the needs program.

In the present paper, we describe the results of a satisfaction survey after participating in a preceptorship program focused on supporting and training healthcare workers from Ghana. All participating organizations contributed substantial time and effort with unique and critical leadership support to enable the success of the program. We acknowledge the countless hours of work, planning, and training from hundreds of colleagues that directly contributed to the success of the program. Both participants and preceptors indicated strong satisfaction in the program with a desire for future participation and expansion of the program. Most participants indicated they achieved all on-site objectives of the preceptorship program with confidence in the post-program objectives of implementation at their home institution. Areas for improvement were identified and included long-term metrics of success and translation of training to direct patient care and outcomes.

The ideal model of medical education and training between HIC programs and LMICs is unclear, though several models of past training exist. These range from short in-person and in-country workshops focusing on a specific skill to that of a diploma oriented long-term (e.g., 12-month duration) training program at a foreign institution. Like our program, the Program for Enhanced Training in Cancer (POETIC) program from Massachusetts General Hospital and Beth Israel Deaconess Medical Center partnered with institutions in South Africa, Tanzania, and Rwanda to develop a 3-week program supplementing training of seven African oncologists practicing in their home countries [15]. Program strengths felt to influence patient care in Africa included: patient-centered care, clinical trial experience, and observation of collaboration among medical, radiation, and surgical oncologists. While development of a skill or skillsets is a critical aspect of training programs, observation of host institution workflow and organization are equally important for the trainees to adapt and develop processes at their own institution based upon the resources available. In the present program, all participants indicated confidence in developing specific guidelines, protocols and workflow for their organization based on their experience with the multi-disciplinary team at Mayo Clinic, which will be presented in a future manuscript.

Program sustainability as well as defined and achievable metrics of success and program sustainability are critical to the long-term success of a training program. Two collaborative head and neck training fellowships at Johns Hopkins and University of Cape Town, which have trained a combined total of 14 trainees, highlight measures of successful clinical training [16]. As a key metric of success, surgical skill transfer and knowledge retention was assessed as measured by the number of head and neck surgical cases, which increased by 335%, in addition to demonstrating increasing complexity level of the procedures. Further, the French African Pediatric Oncology Group has provided clinical training via didactic lectures, both in person and online; onsite practicum; and mentored research projects since 2014 [17]. With nearly 5-years of follow-up that includes 72 participants, a total of four new pediatric oncology units have been established in Niger, Benin, Central African Republic, and Gabon by the graduates of the program. As an example of the importance of sustainability, a genetic testing workshop performed in Kenya in 2013 at the Kenyan National Retinoblastoma Strategy meeting developed an apparent effective program with lectures, group discussions, role play [18]. While the initial metric of success, as defined by a post-workshop assessment, was favorable, within 12 months the post-workshop knowledge matched that of the pre-workshop period.

Our preceptorship program is sustained through a free of charge weekly web-based case-centric lecture series with an emphasis on multi-disciplinary management of cancer. Experts in cancer control, diagnostics, and therapeutics cover a range of critical topics delivered in 6-week blocks of cancer topics relevant to cancer types, stages, and resources available in SSA. With direct input from participants involved in the preceptorship program, the web-based course offerings have been expanded to other disciplines including oncology nursing and medical physics. With over 300 learners in numerous African countries, and more than 100 sessions to date, this model of continuous education has proven highly effective and serves to supplement trainee education at several residency programs in Africa. Additionally, based on a need’s assessment of our African partners, we developed research training and mentorship programs to enhance research capacity and output with our partner institutions.

While many of the reported training programs focus on physician training, a unique aspect of our training program was the inclusion of oncology nurses. A cancer nurse is defined by the European Oncology Nursing Society as a registered nurse with the expert skills, clinical competency, and authority to provide essential nursing care to those with cancer [19]. With greater recognition of the importance of nursing staff, there are now formal development of oncology nurse programs in 16 countries in sub-Saharan African with various levels of training [20]. Many challenges constrain the expansion of oncology nurse training programs, primarily related to scant financial support and lack of recognition of the importance within Africa, as indicated by the fact that less than a third of SSA countries have programs [21, 22]. Training of oncology nurses is a key focus of our program, and in addition to our preceptorship program, we have ongoing continued medical education programs for oncology nurses in Ghana, which focus on safety in administration of anticancer therapies, adverse event management, and palliative care.

In addition to the participant benefits we report in the present study, our survey also revealed many benefits for the participating preceptors. Despite busy clinics and workloads, all preceptor respondents indicated they found joy and value in participating in the program and the desire to participate in future similar programs. The concept of volunteering in global medicine programs to improve mental health and reduce burnout syndrome is not new, and our data may add further evidence that such volunteer programs may be an effective means or strategy to improve both career satisfaction and benefit underserved communities around the globe [23]. It is worth noting that the rate of response from preceptors was less than 50%, which may introduce bias into the analysis and limit our understanding.

The opportunities to collaborate with and support medical colleagues and patients in LMIC are numerous and readily available in all facets of oncology. Here we present the results of participant and preceptor satisfaction in a program that collaborates with academic centers, local governments, and NGOs. With an aging population and soaring cancer rates around the globe, there is an immediate and critical need to broaden our reach and deepen our collaborations to find the means necessary to bridge the ever-widening divide of health disparity.

### Supplementary Information

Below is the link to the electronic supplementary material.

Supplementary file1 (DOCX 31 KB)

## Data Availability

The data supporting the findings of this study are available upon reasonable request from the corresponding author.
