# Supplementary material for: Participant Evaluation of a Multi-disciplinary Oncology Preceptorship Training Program for Oncology Health Professionals from Kumasi, Ghana
Source: J Cancer Educ. 2024 Mar 20;39(4):360–7. doi: 10.1007/s13187-024-02417-w (PMC11219391; doi:10.1007/s13187-024-02417-w)
Supplement: Supplementary file 1 — Supplementary file1 (DOCX 31 KB) [file 13187_2024_2417_MOESM1_ESM.docx]

**Supplemental Table 1.**

**Mayo Scientific Visitor Experience Survey**

Q1 The goals and objectives of the visit were clear

- strongly agree (1)
- Somewhat agree (2)
- Neither agree nor disagree (3)
- Somewhat disagree (4)
- Strongly disagree (5)

Q2 I found the visit to Mayo Clinic valuable and applicable to my clinical practice

- Strongly agree (1)
- Somewhat agree (2)
- Neither agree nor disagree (3)
- Somewhat disagree (4)
- Strongly disagree (5)

Q3 I was able to learn how the multidisciplinary teams treating breast/gynecologic cancer at Mayo Clinic function as a team and as individual specialists.

- strongly agree (1)
- Somewhat agree (2)
- Neither agree nor disagree (3)
- Somewhat disagree (4)
- Strongly disagree (5)

Q4 I was able to review effective and critical elements in the development and expansion of MDT, including team structures, culture, leadership, and context

- True (1)
- False (3)

Q5 I was able to solve practical clinical cases as a team, facilitated by experienced staff from hosting institutions

- True (1)
- False (3)

Q6 I was able to develop ideas and guidelines "share the way forward" for future development in Kumasi.

- strongly agree (1)
- Somewhat agree (2)
- Neither agree nor disagree (3)
- Somewhat disagree (4)
- Strongly disagree (5)

Q11 Please describe the highlights of your trip

________________________________________________________________

Q12 What are the most important takeaways from the trip

________________________________________________________________

Q13 What are areas that we can improve for future trips

________________________________________________________________

**Mayo Clinic Preceptor Experience Survey**

Q1 I believe the training I provided will impact or influence patient care in Ghana?

- Strongly agree (1)
- Somewhat agree (2)
- Neither agree nor disagree (3)
- Somewhat disagree (4)
- Strongly disagree (5)

Q2 I found value or joy in providing training to health professionals from Ghana?

- strongly agree (1)
- Somewhat agree (2)
- Neither agree nor disagree (3)
- Somewhat disagree (4)
- Strongly disagree (5)

Q3 I would like to participate in future opportunities to provide training either at Mayo Clinic or abroad?

- strongly agree (1)
- Somewhat agree (2)
- Neither agree nor disagree (3)
- Somewhat disagree (4)
- Strongly disagree (5)

Q4 I have past experience training health care workers from low- and middle-income countries.

- yes (1)
- No (2)

Q5 Please provide any positive aspects of your experience providing training to health professionals from Ghana.

________________________________________________________________

Q6 Please provide any negative aspects of your experience providing training to health professionals from Ghana

________________________________________________________________

Q7 Please provide any other feedback or recommendations to enhance or improve the training program.

________________________________________________________________
